# PROMBot-HSM-AF: protocol for a feasibility study of a chatbot-based platform to collect patient-reported outcomes after atrial fibrillation ablation

**DOI:** 10.3389/fcvm.2026.1755777

**Published:** 2026-09-02

**Authors:** Sérgio Laranjo, Pedro Silva Cunha, Federico Guede-Fernández, Eduarda Oliosi, Rita Contins, Pedro Dias, Catarina Silva, Ana Clara Félix, Mário Martins Oliveira, Ana Rita Londral

**Affiliations:** 1 Arrhythmology, Pacing and Electrophysiology Unit, Cardiology Service, Santa Marta Hospital, Lisbon, Portugal; 2 Department of Physiology, NOVA Medical School, NOVA University of Lisbon, Lisbon, Portugal; 3 Comprehensive Health Research Center (CHRC), Nova Medical School, NOVA University of Lisbon, Lisbon, Portugal; 4 Physiology Institute, Faculty of Medicine, University of Lisbon, Lisbon, Portugal; 5 Value for Health Collaborative Laboratory, Lisbon, Portugal; 6 Laboratory for Instrumentation, Biomedical Engineering and Radiation Physics (LIBPhys), NOVA School of Science & Technology, NOVA University of Lisbon, Caparica, Portugal; 7 Faculty of Sport (CIAFEL), University of Porto, Porto, Portugal

**Keywords:** atrial fibrillation, catheter ablation, chatbot, conversational agent, feasibility study, patient-reported outcomes, remote patient monitoring

## Abstract

**Introduction:**

Atrial fibrillation (AF) is the most common sustained cardiac arrhythmia, with catheter ablation increasingly recommended as first-line therapy. Despite its effectiveness, 15% to 20% of patients require reassessment within the first month post-procedure. Standard follow-up relies on scheduled consultations and intermittent monitoring, which may delay recognition of recurrence or complications. Remote patient monitoring through conversational agents may address these gaps, yet evidence on feasibility and implementation in post-ablation care remains limited. This study aims primarily to evaluate the feasibility, usability, and acceptability of PROMBot-HSM-AF (Chatbot Platform to Collect Patient-reported Outcomes After AF Ablation, Santa Marta Hospital) during the first 3 months following AF catheter ablation.

**Methods and analysis:**

PROMBot-HSM-AF is a single-center, open-label, randomized controlled pilot (feasibility) trial with two parallel groups, conducted at Santa Marta Hospital, Lisbon, Portugal. Seventy-six patients undergoing AF ablation will be randomized in a 1:1 ratio to either chatbot-supported follow-up or standard care. The primary feasibility parameters are: recruitment rate (proportion of eligible patients consenting), retention rate at 3 months, and chatbot engagement rate (proportion of chatbot prompts answered). Acceptability and usability will be assessed using behavioral indicators (engagement and retention), the System Usability Scale (SUS), the Post-Study System Usability Questionnaire (PSSUQ), and semi-structured interviews with patients and healthcare professionals. Quantitative data will be analyzed descriptively with exploratory between-group comparisons. Semi-structured interviews with patients and healthcare professionals will be analyzed using thematic content analysis.

**Conclusions:**

This pilot trial will provide preliminary evidence on the feasibility and acceptability of integrating a chatbot-based platform into routine post-ablation care. Findings will inform refinement of the intervention and future effectiveness research in this population. The study addresses a critical gap in remote patient monitoring for post-procedural care.

**Clinical Trial Registration:**

https://clinicaltrials.gov/study/NCT07237178, identifier NCT07237178.

## 1 Introduction

Atrial fibrillation (AF) is the most prevalent sustained cardiac arrhythmia, affecting 2% to 4% of the global population (1, 2). Prevalence increases with age, and projections estimate that up to 17.9 million individuals in Europe will be affected by 2060 (3, 4). AF is strongly associated with cardiovascular comorbidities including hypertension, diabetes mellitus, and heart failure, and increases the risk of stroke fivefold and all-cause mortality twofold (3, 5). These epidemiological trends underscore the need for scalable and sustainable strategies to optimize AF management.

Catheter ablation is increasingly utilized as first-line therapy in selected patients, as recommended by the European Society of Cardiology and the American College of Cardiology/American Heart Association guidelines (6). While ablation effectively improves symptoms and quality of life, a substantial proportion of patients experience recurrence or complications requiring reassessment. Studies indicate that 15% to 20% of patients require evaluation within the first month post-ablation, and up to 45% within the first year (7, 8). Current follow-up strategies predominantly rely on scheduled in-person consultations and intermittent monitoring, approaches that may delay recognition of recurrence or complications (9–11).

Remote patient monitoring (RPM) has emerged as a promising strategy to enhance continuity of care and enable early detection of clinical deterioration (12, 13). RPM encompasses telemonitoring, telemedical interventions, and structured patient education delivered through digital platforms (14). Systematic reviews support the effectiveness of RPM in improving cardiovascular outcomes, though effect sizes vary across populations and intervention designs (15). Conversational agents, commonly referred to as chatbots, represent a specific modality of RPM that may facilitate behavior change and increase patient engagement across diverse populations and age groups (16). In cardiovascular care, recent studies have demonstrated promising results, for instance, Fukuoka et al. (17) reported increased awareness of myocardial infarction symptoms among women using a chatbot intervention, and Trivedi et al. (18) documented high engagement rates with artificial intelligence-driven follow-up telephone calls after AF hospitalization.

Despite these advances, several critical knowledge gaps persist. First, evidence regarding the implementation of RPM specifically in post-procedural contexts, including after AF catheter ablation, remains limited (19). Second, although RPM has been associated with improvements in functional status and reduced acute care utilization, its effects on broader clinical outcomes and health-related quality of life remain inconclusive (20). Third, current European guidelines highlight gaps in patient-centered management, particularly regarding the role of RPM and telemedicine in AF care (21). Successful adoption of RPM requires not only appropriate technology but also coordinated multidisciplinary healthcare teams, robust patient engagement strategies, and integration into existing clinical workflows (22, 23).

These gaps underscore the need for studies evaluating co-created, patient-centered digital interventions that systematically integrate patient-reported outcome measures (PROMs) into AF follow-up care. The PROMBot-HSM-AF (Chatbot Platform to Collect Patient-reported Outcomes After AF Ablation, Santa Marta Hospital) was developed through a participatory co-creation process involving cardiologists, nurses, patients, engineers, and health scientists to address these needs and optimize the feasibility of real-world implementation. The platform is delivered through WhatsApp, leveraging a widely adopted messaging service with 73% penetration in Portugal overall and 62% among adults aged 55 to 64 years (24). Unlike standard follow-up, which offers limited early post-ablation contact and no routine PROM capture, structured digital PROM collection has been associated with earlier detection of arrhythmia recurrence in comparable post-ablation populations (13), suggesting potential to complement existing care through more timely clinical response.

The primary objective of this pilot study is to evaluate the feasibility and acceptability of integrating PROMBot-HSM-AF, a chatbot-based platform for collecting PROMs and clinical parameters, into routine post-ablation follow-up care at a tertiary hospital. The specific aims, framed for the feasibility and pilot phase, are to: (1) quantify recruitment, retention, and engagement rates over the 3-month follow-up and compare each against pre-specified progression thresholds (retention ⩾80%, engagement ⩾70%), constituting the primary outcomes; and, as secondary, exploratory outcomes, (2) assess the usability and acceptability of the chatbot (patients) and of the clinical monitoring interface (healthcare professionals) at 3 months, using validated usability measures (System Usability Scale [SUS], Post-Study System Usability Questionnaire [PSSUQ]), behavioral indicators, and semi-structured interviews; and (3) evaluate the preliminary integration and data completeness of PROMs (e.g., Atrial Fibrillation Effect on Quality-of-Life [AFEQT]) collected via the platform over the 3-month follow-up, and explore between-group differences as hypothesis-generating estimates to inform a definitive trial.

## 2 Methods and analysis

### 2.1 Study hypotheses

We hypothesize that integrating PROMBot-HSM-AF into routine post-ablation follow-up care will be feasible and acceptable, and that structured, high-frequency remote data collection will be associated with improved patient engagement in follow-up care and more favorable trends in health-related quality of life (AFEQT) relative to standard care.

### 2.2 Study design

**Figure 1:**
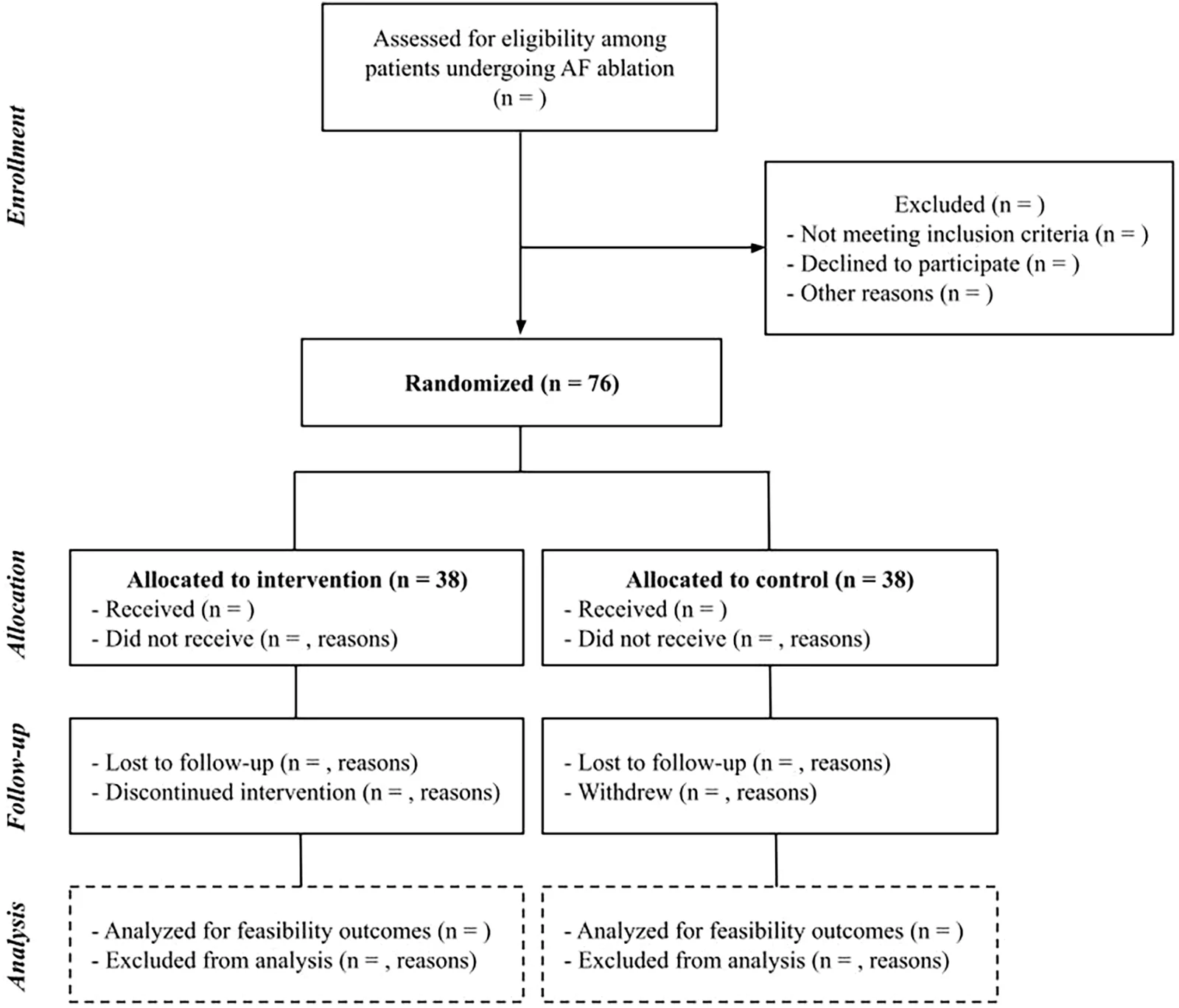
Flowchart diagram for the study PROMBot-HSM-AF.

This study is a single-center, open-label, randomized controlled pilot (feasibility) trial with two parallel groups. Patients undergoing catheter ablation for AF will be randomly assigned 1:1 to chatbot-supported follow-up via the PROMBot-HSM-AF platform (intervention) or standard care (control). The control arm comprises structured discharge education and scheduled 1- and 3-month appointments without remote monitoring between visits (detailed under Procedures). The study flow diagram (Figure 1) represents the feasibility pathway.

### 2.3 Study setting

The feasibility study is conducted at the Department of Cardiology, Santa Marta Hospital, Lisbon, Portugal. This tertiary referral center specializes in cardiothoracic care and performs a high volume of cardiac ablation procedures annually. Since 2011, the hospital has implemented a structured 1-year follow-up program involving periodic telephone consultations, creating a strong organizational foundation for digital health integration (25, 26).

Participant recruitment, informed consent procedures, and clinical assessments occur at the hospital. The intervention is delivered remotely, with patient-chatbot interactions occurring in participants’ home environments. Clinical coordination and data monitoring are centralized at the hospital to ensure integration with standard care pathways.

#### 2.3.1 Inclusion criteria

Patients are eligible for enrollment if they meet all of the following criteria:

1. Age 18 years or older;
2. Documented diagnosis of paroxysmal or persistent AF based on clinical assessment and coded according to the International Classification of Diseases, 11th Revision;
3. Undergone catheter ablation for AF at Santa Marta Hospital during the study recruitment period;
4. Able to use the PROMBot-HSM-AF chatbot independently or with caregiver assistance (Portuguese literacy sufficient to follow instructions and ability to operate a smartphone); digital literacy is assessed individually, with caregiver-assisted enrollment available for older adults willing to learn;
5. Clinically stable at discharge, defined as absence of any complication requiring continued inpatient care or urgent reintervention (e.g., cardiac tamponade, major access-site bleeding, high-grade atrioventricular block); minor, self-limiting findings (e.g., uncomplicated groin haematoma) do not preclude participation;
6. Provision of written informed consent.

#### 2.3.2 Exclusion criteria

Patients are excluded if they meet any of the following criteria:

1. Severe cognitive impairment preventing comprehension of study procedures, such as moderate-to-severe dementia or other neurological conditions limiting capacity to consent;
2. Severe uncorrected visual impairment or motor disability preventing smartphone operation even with caregiver support;
3. Explicit refusal to use digital technology or unwillingness to share health data required for the study;
4. Clinical or contextual conditions rendering 3-month follow-up unfeasible, including anticipated relocation outside the hospital catchment area or concurrent participation in another interventional study with overlapping endpoints;
5. Life expectancy less than 6 months.

### 2.4 Sample size

This pilot was sized to estimate feasibility parameters, not clinical efficacy. Using G*Power 3.1, sample sizes were derived from one-sided proportion tests (80% power, α=0.05) for retention and engagement; the one-sided test reflects the directional progression criteria, where only meeting or exceeding the thresholds is relevant. Thresholds were retention ⩾80% and engagement ⩾70%, yielding 62 and 56 participants, respectively; allowing for 20% dropout, the final target was 76 (38 per group). Both the directional hypothesis and the ⩾70% threshold are consistent with adherence benchmarks for feasibility pilots (27) and with retention and engagement rates reported in a comparable cardiovascular RPM intervention (e.g., 71%–94% sustained activity/survey completion over six months) (28).

### 2.5 Randomization and allocation concealment

Participants will be randomly assigned to the intervention or control group using computer-generated random numbers in permuted blocks of variable size (4 to 6) to maintain allocation concealment. Randomization will be stratified by AF type (paroxysmal vs persistent) to ensure balance between groups, as AF type is a recognized prognostic factor for recurrence after catheter ablation (29). Allocation sequences will be generated by a statistician not involved in participant recruitment. Allocation concealment will be maintained through sequentially numbered, opaque, sealed envelopes prepared by personnel independent of the recruitment process, opened only after eligibility confirmation and completion of baseline assessments.

### 2.6 Blinding

Due to the nature of the intervention, it is not feasible to blind participants or healthcare professionals providing care. However, outcome assessors conducting medical record reviews for clinical endpoints (e.g., hospitalizations, cardiovascular events) will be blinded to group allocation where possible. Statistical analyses will be conducted by a researcher blinded to group assignment until completion of primary analyses. Participants will be instructed not to disclose their group allocation during follow-up assessments conducted by blinded assessors.

### 2.7 Procedures

#### 2.7.1 PROMBot-HSM-AF platform intervention

The research team will configure the PROMBot-HSM-AF chatbot and related remote monitoring platform, including definition of finite-state dialogue flows with AF-specific questions, message content, and input-validation rules. Pilot testing with 2–3 test users will also be conducted to identify usability issues and to verify delivery, latency, and data integrity. Health professionals involved in recruitment and follow-up will receive training on study procedures, including informed consent, platform registration, and use of the clinician interface to monitor incoming data.

Eligible patients undergoing AF ablation will be approached during their hospital stay. After receiving written study information, discussing questions, and providing informed consent, participants will complete a baseline assessment that will include demographics, medical history, recent clinical parameters (e.g., blood pressure and heart rate), and health-related quality of life (AFEQT, European Portuguese version) (13, 30). For participants randomized to the intervention group, PROMBot-HSM-AF will be set up before discharge by confirming WhatsApp is installed on the participant’s smartphone, registering the participant in the remote monitoring platform, linking their mobile number to PROMBot-HSM-AF, and sending a confirmatory message to verify correct enrollment. Patients (and caregivers, if applicable) will receive a brief demonstration and training, delivered by the study nurse, to ensure they can interact with the chatbot autonomously, and will also receive a leaflet with a brief explanation of the chatbot.

Participants in the intervention group will engage with PROMBot-HSM-AF via chatbot for 12 weeks. The chatbot will request self-reported health metrics (e.g., body mass, blood pressure, heart rate, step count, AF-related symptoms) and general well-being, supplemented with brief educational prompts (31). Participants who do not already own a validated blood pressure monitor will be provided with one at enrollment; device ownership is assessed during the baseline visit.

In the first month, chatbot interactions will occur daily, focusing on post-ablation symptom surveillance and medication adherence. After that, the cadence will taper to reduce burden in later weeks while maintaining essential monitoring. Chatbot prompts will remain open for 24 h, after which nonresponses will be recorded as missing data. A study nurse or physician will review patient entries weekly and may proactively contact participants reporting concerning symptoms or lapses in engagement. PROMBot-HSM-AF will not replace routine follow-up; all patients will continue standard clinical care, with chatbot data available to the study clinical team to complement or inform consultations at the treating cardiologist’s discretion.

At the end of the 3-month follow-up period, all participants will undergo post-intervention assessments during a routine hospital visit. If necessary, these assessments will be conducted via secure videoconference. The PROMs will focus on the effect of AF on health-related quality of life. Participants in the intervention group will also complete validated measures of usability and perceived usefulness. They will also take part in a brief semi-structured interview to share their experience of using the chatbot. Semi-structured interviews will also be conducted with healthcare professionals directly involved in patient care (e.g., cardiologists, nurses, and technicians). These interviews are intended to elicit qualitative insights regarding their experience with the chatbot-based intervention, as well as their perceptions of its usability, clinical relevance, and impact on workflow integration.

#### 2.7.2 Control group

Participants allocated to the control group receive standard post-ablation care as delivered at Santa Marta Hospital, which includes structured discharge instructions with written educational materials about post-ablation care, scheduled follow-up appointments at 1 month and 3 months post-discharge, open access to the cardiology clinic telephone line during working hours for clinical concerns, and a 24-h hospital telephone service for urgent medical issues. Control group participants do not receive remote monitoring between scheduled appointments.

Both intervention and control groups complete identical standardized assessments at baseline (pre-discharge) and 3 months post-ablation. The timing of data collection activities is outlined in Table 1.

**Table 1:** Schedule of enrollment, interventions, and assessments for the PROMBot-HSM-AF trial.

TRIAL PERIOD		Enrollment	Post-randomization				
Timepoint	Arm	⩽ day 0	Day 0	M1	M2	M3	M12
Target window		up to day 0	day 0	30±3 d	60±5 d	90±5 d	365±14 d
ENROLLMENT							
Eligibility screen	I+C	X					
Informed consent	I+C	X					
Randomization/allocation	I+C		X				
INTERVENTIONS							
PROMBot-HSM-AF	I			X	X	X	
Standard care	C		X	X	X	X	
PRIMARY OUTCOMES (FEASIBILITY)							
Recruitment rate	I+C	X	X				
Retention rate	I+C					X	
Engagement rate	I			X	X	X	
BASELINE CHARACTERISTICS							
Demographics	I+C		X				
AF history & comorbidities	I+C		X				
Procedure-related characteristics	I+C		X				
CLINICAL OUTCOMES (HOSPITAL-REPORTED)							
AF recurrence (AAD/cardioversion/repeat ablation)	I+C			X	X	X	X
Complications (readmission, ED visit, thromboembolism, major bleeding, tamponade)	I+C			X	X	X	X
All-cause mortality	I+C						X
Cardiology/internal-medicine consultations	I+C					X	X
Diagnostic tests (ECG, Holter)	I+C			X	X	X	X
PROMs							
AFEQT	I+C		X			X	
ESC quality-indicator items (weekly, rotating)	I			X	X	X	
Symptom checklist	I			X	X	X	
Medication adherence	I			X	X	X	
BIOSIGNALS							
Body mass	I			X	X	X	
Blood pressure	I			X	X	X	
Heart rate	I			X	X	X	
Step count	I			X	X	X	
ACCEPTABILITY & USABILITY							
Patient experience (SUS, PSSUQ, interview)	I					X	
Healthcare-professional experience (SUS, PSSUQ, interview)	HCP					X	

The schedule follows the SPIRIT figure convention (34).

M, month; d, days. Arm: I = intervention; C = control; HCP = healthcare professionals.

#### 2.7.3 Clinical monitoring interface

Healthcare professionals access patient data through a secure web-based clinical monitoring interface integrated with the hospital’s electronic health record system. The interface provides real-time visualization of patient-reported data with trend graphs, automated alerts for concerning values, patient engagement metrics, and summaries of symptom reports.

A designated study nurse reviews the interface weekly during routine working hours. The clinical team maintains 24-h access for clinical decision-making purposes. All access is logged for audit purposes.

#### 2.7.4 Safety monitoring protocol

Automated alerts are generated when patient-reported values exceed predefined clinical thresholds or when concerning symptom patterns are identified. Alert thresholds are defined as follows:

**Critical alerts (immediate response required):** Systolic blood pressure ⩾180 mmHg or less than 90 mmHg; diastolic blood pressure greater than 110 mmHg or less than 60 mmHg (32, 33); heart rate greater than 120 bpm or less than 50 bpm (29); new onset chest pain, syncope, or severe dyspnea; or three consecutive missed responses indicating potential disengagement.

**Response protocol for critical alerts:** The study nurse contacts the participant by telephone within 1 h during working hours or within 4 h outside working hours. For immediately life-threatening symptoms (e.g., chest pain suggestive of acute coronary syndrome, syncope), participants are advised to contact emergency medical services. Before escalation, the nurse verifies implausible values with the participant (confirming the technique and requesting a repeat reading) to exclude measurement error, escalating conservatively where uncertainty persists. For other concerning values, the study nurse coordinates with the treating cardiologist to determine appropriate clinical action, which may include scheduling an urgent clinic appointment, ordering diagnostic tests, or adjusting medications.

**Warning alerts (review within 24 h):** Systolic blood pressure 160 to 179 mmHg or diastolic blood pressure 100 to 109 mmHg on two or more occasions(32); heart rate 100 to 119 bpm or 50 to 60 bpm on two or more occasions(29); or reported worsening of existing symptoms.

All alerts and clinical actions taken in response are documented in both the study database and the participant’s electronic health record. Participants receive clear written instructions at enrollment specifying symptoms requiring immediate emergency medical attention vs. those that can be reported through the chatbot for routine clinical follow-up.

### 2.8 Technical description of the chatbot

**Figure 2:**
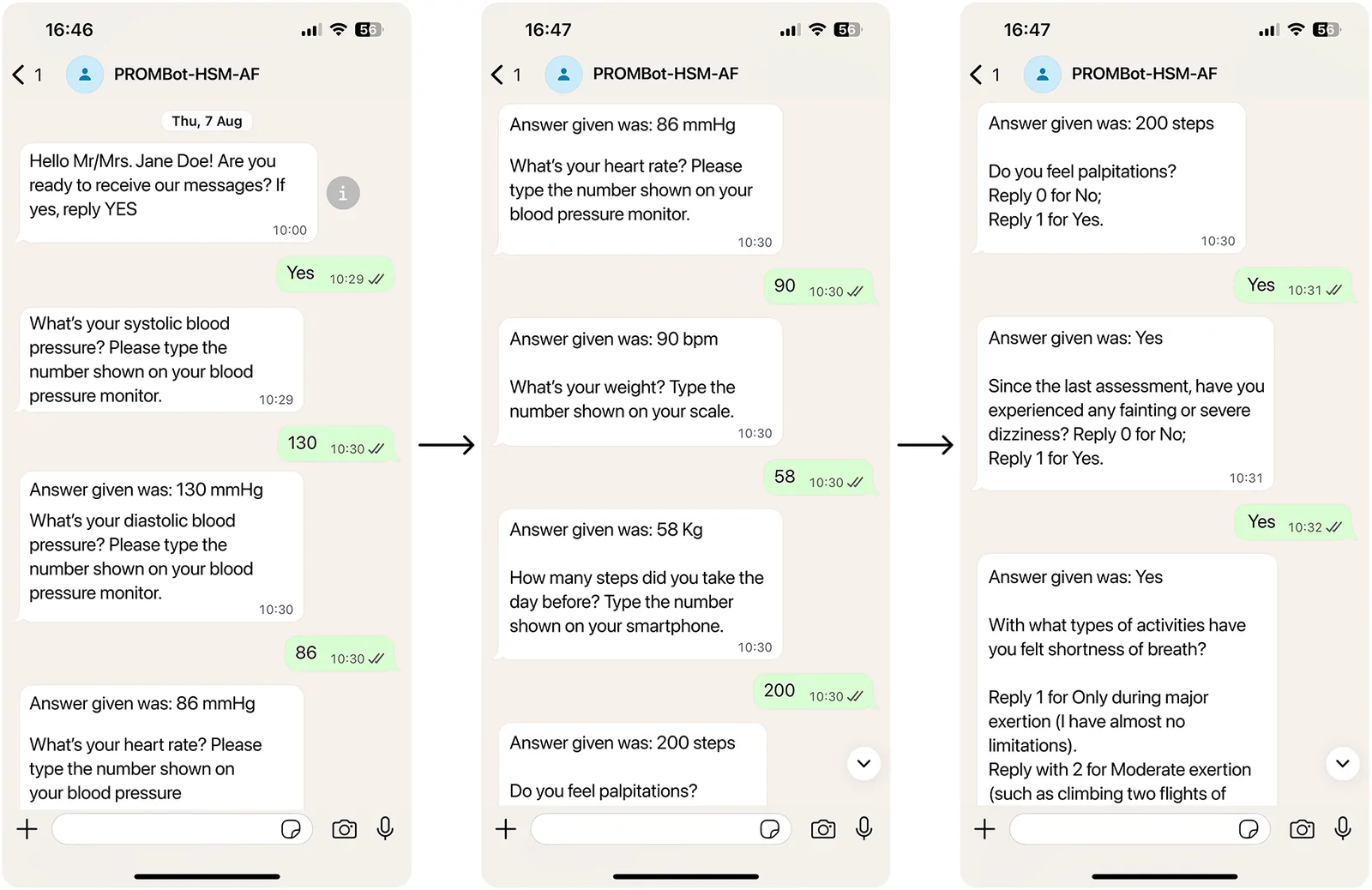
Example screenshots of the PROMBot-HSM-AF interface.

The PROMBot-HSM-AF chatbot is a rule-based, finite-state-machine chatbot delivered via WhatsApp, supporting numeric, multi-choice and free-text inputs with branching and input-validation rules. Unlike standard follow-up, it enables structured, time-stamped collection of PROMs and self-measured physiological data between clinic visits, with conditional follow-up questionnaires triggered by specific symptoms to provide clinicians with additional detail. Collected measures, their frequency, and timing are detailed in Table 1 (34); Figure 2 illustrates an example WhatsApp conversation flow. The chatbot operates in European Portuguese, with screenshots shown in English for illustrative purposes; technical terminology (e.g., systolic/diastolic blood pressure) is explained in plain language during patient training at enrollment. Full implementation details are provided in our previously published work (35).

### 2.9 Outcome measures

Data from participating patients will be systematically collected and analyzed to evaluate predefined study outcomes. Outcomes are organized as a single set of primary feasibility outcomes and several sets of secondary or exploratory outcomes grouped by data type and source; baseline variables are reported as descriptors. The study arm and timepoints for every measure are summarized in Table 1.

#### 2.9.1 Primary outcomes (feasibility)

The primary outcomes of this trial are feasibility parameters, including recruitment rate (eligible patients providing consent), retention rate (participants completing the 3-month follow-up), and engagement rate (proportion of chatbot prompts answered within 24 h) (36, 37).

#### 2.9.2 Baseline characteristics

Baseline characteristics included year of birth, sex, body mass, highest level of education, and current city of residence.

#### 2.9.3 Clinical outcomes

Clinical outcomes include AF recurrence, defined as documented symptomatic relapse or the requirement for antiarrhythmic therapy, cardioversion, or repeat ablation, assessed within three months and through twelve months post-ablation. Post-ablation complications comprise unplanned hospital readmissions, emergency department visits, thromboembolic events, major bleeding, or cardiac tamponade. Additional outcomes are all-cause mortality within twelve months, the number of cardiology or internal medicine consultations within three and twelve months (routine or symptom-driven), and additional diagnostic tests (e.g., ECG, Holter monitoring) performed during follow-up. Clinical data will be obtained from hospital records and complemented by baseline information on AF history, comorbidities, and procedure-related characteristics.

#### 2.9.4 Patient-reported outcomes and biosignals

Health-related quality of life will be assessed using the AFEQT, a validated, disease-specific instrument for AF (38). The AFEQT comprises 20 items across four domains (Symptoms, Daily Activities, Treatment Concerns, and Treatment Satisfaction) with an overall score (0–100) derived from the first three domains; higher scores indicate better quality of life. A change of ⩾5 points is generally considered clinically meaningful (13). The AFEQT will be administered at baseline and follow-up to assess changes in AF-related symptoms and the broader impact of management strategies on daily life. To complement the AFEQT, participants will complete brief, rotating weekly questions aligned with the ESC quality of treatment indicators (31), assessing symptom status, physical functioning, emotional well-being, cognitive function, and knowledge of AF-related risk factors. Each question will be administered once per week to minimize participant burden.

All PROMs and biosignals will be collected via the chatbot. Body mass, blood pressure, and heart rate will be self-reported daily for the first four weeks and weekly thereafter, with blood pressure and heart rate measured using a blood pressure monitor. Physical activity will be tracked as daily step counts via the smartphone pedometer. Symptoms, including palpitations, dyspnea, chest pain, dizziness/syncope, fatigue, puncture-site changes, and edema, will also be reported daily during the first four weeks and weekly thereafter. To mitigate self-report bias, participants receive standardized training on device use and measurement technique at enrollment.

#### 2.9.5 Chatbot system usability and perceived usefulness

The usability and acceptability of the system will be assessed using the System Usability Scale (SUS), a widely adopted 10-item self-administered questionnaire that generates a global usability score ranging from 0 to 100. Scores above 68 are generally interpreted as reflecting good usability. The European Portuguese version has been translated, culturally adapted, and validated, demonstrating good construct validity through significant correlations with the PSSUQ (r=0.70) and with a general usability item (r=0.48) (39). User satisfaction and perceived usefulness will be evaluated using the Post-Study System Usability Questionnaire (PSSUQ), a 19-item scale validated in European Portuguese, which has shown excellent internal consistency (a=0.80), satisfactory inter-rater reliability (ICC = 0.67), and strong construct validity (r=0.84, P<.05) (40). In this instrument, lower scores indicate greater satisfaction. Both SUS and PSSUQ will be administered at follow-up to provide a comprehensive evaluation of participants’ (patients and health professionals) experiences with the PROMBot-HSM-AF intervention.

To complement quantitative assessments, semi-structured interviews will be conducted with patients (see Supplementary Table S1) to qualitatively explore their experiences with PROMBot-HSM-AF. Healthcare professionals directly involved in patient care (e.g., cardiologists, nurses, and technicians) will also participate in semi-structured interviews (see Supplementary Table S1) to provide insights into usability, workflow integration, perceived utility, time burden, and potential improvements.

### 2.10 Data analysis

This study will employ a mixed-methods approach, combining quantitative and qualitative data from both patients and health professionals, to enable a comprehensive evaluation of the intervention’s outcomes and implementation processes, consistent with recommended mixed-methods approaches for feasibility trials (27).

#### 2.10.1 Quantitative data

Participant characteristics and study outcomes will be summarized using descriptive statistics. Continuous variables (e.g., age, body mass, blood pressure) will be reported as means ± SDs or medians [IQR], depending on distribution, while categorical variables (e.g., sex, educational level, comorbidities) will be expressed as frequencies and percentages. Comparisons between groups will be performed using independent t-tests or Mann-Whitney U tests for continuous variables, and chi-square or Fisher exact tests for categorical variables, with 95% confidence intervals reported. The effect of the intervention on primary and secondary AF-related outcomes will be examined using regression models with and without adjustment for baseline covariates. Formal hypothesis testing is not the primary focus. All analyses will be conducted in R (version 4.5.0), with statistical significance defined as a two-sided p-value <0.05.

#### 2.10.2 Qualitative data

Qualitative data related to the process and outcomes of the intervention will be analyzed using thematic content analysis. The analysis framework will integrate topics from the semi-structured interview guide with themes that emerge inductively during transcript familiarization. All interviews will be audio-recorded and transcribed verbatim using transcription software to capture participants’ responses. Two researchers will independently code the data, with discrepancies resolved through discussion to ensure coding reliability. Key themes will be illustrated with anonymized quotations. A purposive subsample of participants (10-15 from the intervention arm) (41, 42) will be interviewed to gain in-depth insights into usability, engagement, and perceived outcomes of the intervention.

#### 2.10.3 Patient and public involvement

Patients with lived experience of AF and ablation procedures contributed to the design of PROMBot-HSM-AF during the developmental phase. Patient representatives participated in focus groups to inform chatbot dialogue flows, educational content, and selection of PROMs prioritized by patients. Patients were not involved in the development of this study protocol but will be invited to participate in dissemination activities, including co-authorship of plain-language summaries for patient organizations.

## 3 Discussion

This protocol describes the design of PROMBot-HSM-AF, a feasibility randomized controlled trial evaluating the feasibility and acceptability of a chatbot-based platform for RPM after AF catheter ablation. This study addresses critical gaps in post-procedural surveillance by embedding structured collection of PROMs and clinical parameters into an accessible, widely adopted communication platform. The co-creation approach involving multidisciplinary stakeholders, including patients, cardiologists, nurses, engineers, and health scientists, distinguishes this intervention from commercially available applications that often lack clinical validation and integration with healthcare workflows (43).

The selection of a chatbot-based delivery platform merits consideration. App-based digital health interventions frequently encounter barriers to adoption related to installation requirements, user account creation, and ongoing technical maintenance (44). WhatsApp’s ubiquitous presence in Portugal, with particularly high penetration among middle-aged and older adults (24), may reduce these barriers and enhance reach to populations disproportionately affected by AF. Furthermore, integration with existing communication behaviors may promote sustained engagement compared with standalone health applications requiring users to remember to open a separate app (45).

The PROMBot-HSM-AF intervention builds upon and extends previous digital health research in AF populations. Misra et al. (46) demonstrated the feasibility of a 12-week remotely supervised exercise and education program for patients post-ablation, achieving high adherence rates and improvements in body mass, blood pressure, physical activity levels, and AFEQT scores. The PROMBot-HSM-AF platform similarly aims to support self-monitoring and self-reporting but embeds PROM collection into routine monitoring rather than requiring participation in a structured behavioral intervention program, potentially enhancing scalability. However, patient training relied on a dedicated study nurse, and resourcing this role in routine care will be important for future scale-up.

The emPOWERD-AF trial evaluated the MyAlgos platform, an application-based disease management system, and reported improvements in quality of life and favorable usability ratings from physicians (47). However, application-based approaches require installation and ongoing engagement with a standalone digital product. PROMBot-HSM-AF’s integration with WhatsApp may enhance accessibility, particularly for individuals with limited digital literacy or those reluctant to install additional health applications. Research on conversational agents in cardiovascular care has demonstrated promise. Guhl et al. (48) found that a relational agent intervention improved quality of life and anticoagulation adherence among patients with AF. More recently, Trivedi et al. (18) demonstrated high engagement with artificial intelligence-driven follow-up telephone calls after AF hospitalization, with positive effects on medication adherence and patient experience. PROMBot-HSM-AF synthesizes elements from these approaches, i.e., systematic data collection, educational support, and rule-based interactive prompts, while prioritizing rigorous feasibility assessment before scaling. This design is deliberate, with clinical escalation managed by the study team rather than the chatbot.

A critical gap addressed by PROMBot-HSM-AF is the focus on post-procedural monitoring during the highest-risk period for complications. While chronic disease management applications abound, evidence regarding digital monitoring specifically after cardiac procedures remains limited (11). The immediate post-ablation period presents distinct surveillance challenges, including distinguishing expected post-procedural symptoms from concerning complications, managing the blanking period during which arrhythmia recurrence is common and often transient, and supporting adherence to new or modified medication regimens. PROMBot-HSM-AF’s graduated monitoring schedule, intensive daily surveillance in month 1 tapering to weekly monitoring in months 2 and 3, reflects these evolving needs. Continuous self-monitoring may also heighten anxiety in AF patients (49). This risk is addressed through educational content and the informed-consent process, with reassurance regarding the expected, transient nature of early post-ablation symptoms.

This study has several strengths. The co-creation process engaged stakeholders throughout intervention development, enhancing relevance to patient needs and clinical workflows. The mixed-methods design will provide nuanced understanding of implementation facilitators and barriers beyond feasibility metrics alone. Explicit predefinition of feasibility success criteria enables transparent interpretation of whether progression to a definitive trial is warranted. The pragmatic setting in a high-volume tertiary hospital enhances external validity to similar clinical contexts.

Several limitations merit acknowledgment. As a single-center feasibility study, findings may not generalize to healthcare systems with different organizational structures, digital infrastructure, or patient populations. The open-label design introduces potential for observation bias, though blinding of outcome assessors for objective clinical endpoints will partially mitigate this concern. The 3-month follow-up duration, while justified by the post-ablation blanking period convention, may not capture delayed recurrences or later medication transitions (e.g., discontinuation of antiarrhythmic or anticoagulant therapy).

Consistent with its feasibility design, comparisons of clinical outcomes are exploratory and should not be interpreted as evidence of effectiveness. Requiring digital literacy and Portuguese-language smartphone use introduces socio-digital exclusion, favoring younger, more digitally engaged patients over older, lower-socioeconomic patients—those with the highest AF burden yet least able to sustain digital engagement (50). Findings may therefore generalize primarily to digitally literate patients, and PROMBot-HSM-AF is intended to complement, rather than replace, usual care, with equity-oriented adaptations warranting evaluation before scale-up. Reliance on self-reported measurements introduces measurement error, including potential adherence or reporting bias (e.g., avoidance of unfavorable readings); validated devices and standardized training at enrollment will partially mitigate, though not eliminate, this risk, and no formal calibration or cross-validation against clinically measured values was performed within the study protocol. Finally, intensive daily monitoring may itself heighten symptom awareness and care-seeking, introducing a potential Hawthorne effect (51) to be considered when interpreting feasibility outcomes.

If predefined feasibility criteria are met, findings from this pilot will inform the design of a definitive multi-center randomized controlled trial powered to detect clinically important differences in AF recurrence, complications, quality of life, and healthcare utilization. A full economic evaluation, including cost-effectiveness and budget impact analyses, would be incorporated to assess sustainability and inform health policy decisions. The qualitative findings will guide refinement of the intervention, particularly by optimizing alert thresholds, chatbot dialogue flows, and clinical monitoring interface features to minimize alert fatigue while maintaining safety, and by informing a risk-stratified approach that targets intensive digital monitoring to patients most likely to benefit (e.g., those with educational needs, poor symptom control, or elevated recurrence risk) rather than applying it uniformly.

If feasibility criteria are not met, qualitative data will illuminate specific barriers to implementation. Potential adaptations might include reduced prompt frequency, enhanced training protocols, integration with alternative messaging platforms if WhatsApp engagement proves insufficient, or simplified data collection focused on a core set of essential parameters. The modular design of PROMBot-HSM-AF enables iterative refinement of individual components based on empirical findings.

Beyond the immediate AF post-ablation context, findings may inform broader applications of chatbot-based monitoring in post-procedural care across cardiovascular and other medical specialties. The safety monitoring protocols and workflow integration strategies developed here may serve as templates for similar interventions. Furthermore, the structured PROMs and biosignal data collected through this platform will contribute to the CardioCausalAI project’s development of causal artificial intelligence models for predicting complications and optimizing clinical decision-making (48).

## 4 Ethics and dissemination

The hospital ethics committee approved the study protocol (Ethics Committee approval number 974/2020). All participants will provide written informed consent before enrollment. The study will be conducted in accordance with the Declaration of Helsinki and complies with the European Union General Data Protection Regulation (GDPR). Participants may withdraw from the study at any time without impact on their clinical care.

The PROMBot-HSM-AF platform was deployed on Heroku, with application data stored in a Heroku Postgres database hosted in the European region. Communication between participants and the platform was implemented through Twilio’s WhatsApp Business API. The infrastructure incorporated technical safeguards such as encrypted connections, access-controlled databases, restricted access to the study dashboard, and provider security controls. However, the use of WhatsApp introduced additional ethical and data protection considerations because the intervention involved the collection of health-related information, including physiological measures, symptoms, medication adherence, quality of life, well-being, physical function, and adverse-event-related responses. Although the research team controlled the study database and application backend, message transmission depended on third-party providers, including Twilio and Meta/WhatsApp. As a result, message content and metadata, such as phone numbers, timestamps, delivery information, and technical routing data, could be processed outside the research team’s full control.

The main risks identified were third-party processing of health-related content or metadata and sub-processing or international transfer arrangements. Accordingly, the relevant WhatsApp and Twilio data processing terms were reviewed by a data protection legal expert, who confirmed the associated third-party data processing risk. These risks were mitigated through data minimization, use of mostly structured questions, avoidance of open questions, restricted access to the study database and dashboard, and explicit information to participants during informed consent.

Data will be pseudonymized throughout the trial, with data access limited to authorized study personnel. The trial is registered at ClinicalTrials.gov with the identifier: NCT07237178.

This study is conducted within the scope of the CardioCausalAI project, funded by the Foundation for Science and Technology, Portugal (reference 2024.07369.IACDC), which develops causal artificial intelligence models for cardiovascular care.

Study findings will be disseminated through peer-reviewed publications in open-access journals, presentations at national and international cardiology and digital health conferences, and knowledge translation activities targeting clinical stakeholders and health policy audiences. A plain-language summary will be prepared for dissemination through patient advocacy organizations. Results will be reported in accordance with the Consolidated Standards of Reporting Trials extension for pilot and feasibility trials (CONSORT-PF) (37) and the Standards for Reporting Qualitative Research (SRQR) guidelines (52).
