# Supplementary material for: PROMBot-HSM-AF: protocol for a feasibility study of a chatbot-based platform to collect patient-reported outcomes after atrial fibrillation ablation
Source: Front Cardiovasc Med. 2026 Sep 2;13:1755777. doi: 10.3389/fcvm.2026.1755777 (PMC13579452; doi:10.3389/fcvm.2026.1755777)
Supplement: Supplementary file 1 [file Table1.pdf]

Supplementary Material

1 SUPPLEMENTARY TABLES

Table S1. Semi-structured interview on the experience of patients and healthcare professionals with PROMBot-FA

**Patients**
• Do you consider PROMBot easy to use?
• Do you consider PROMBot useful? Please answer yes or no.
• What type of messages from PROMBot were the most useful for you?
• What improvements would you make to PROMBot?
• Was interacting with PROMBot fun?
• Would you like to continue using PROMBot to record your values? Why? Please briefly justify your reasons for wanting or not wanting to continue.
• Would you like to continue using PROMBot to receive medication reminders? Why? Please briefly justify your reasons for wanting or not wanting to continue.
• Would you like to continue using PROMBot to receive tips on healthy habits? Why? Please briefly justify your reasons for wanting or not wanting to continue.
**Healthcare professionals**
• Do you consider PROMBot easy to integrate into your clinical context and usual workflow?
• Do you consider PROMBot useful for the follow-up and management of your patients? Please justify.
• What type of messages or functionalities of PROMBot did you find most relevant or effective in clinical practice?
• What improvements would you suggest to PROMBot to increase its effectiveness or facilitate its use by the healthcare team?
• Do you consider interacting with PROMBot to have been a positive or pleasant experience for you as a professional?
• Would you like to continue using PROMBot as a support tool for monitoring your patients? Why?
• Do you consider the use of PROMBot useful for the automatic sending of medication reminders? Please explain your opinion.
• Do you consider it relevant to maintain the sending of lifestyle advice through PROMBot? Please justify your answer.
• Do you estimate that the use of PROMBot required significant additional time in your workday?
• Do you believe that the use of PROMBot contributed to the improvement of care provided? Please explain how.
